# Assessing Ecosystem Services to Identify Conservation Priorities

**DOI:** 10.1371/journal.pbio.0040392

**Published:** 2006-10-31

**Authors:** Liza Gross

Efforts to save wilderness often play out within a win-lose framework, pitting conservation against economic opportunity. But as human pressures on wildlands continue to escalate, conservation biologists are seeking win-win approaches, based on the notion that ecosystems provide numerous economic benefits—wetlands mitigate flooding, for example—to a wide variety of beneficiaries. By quantifying these ecosystem services and the “opportunity” costs of not developing habitat, planners can identify areas that provide important ecosystem services and determine who benefits from these services and who incurs costs. But quantifying costs and benefits and the flow of ecosystem services across a variable landscape is a daunting task. Thus far, it has not been clear to what degree traditional conservation plans for biodiversity also protect valuable ecosystem services.

Taking complementary approaches to this problem, two new studies use spatially explicit models to incorporate ecosystem services into conservation planning. In one study, Robin Naidoo and Taylor Ricketts weigh the economic value of five ecosystem services against the costs of conservation in the Atlantic forests of Paraguay. In the second study, Kai Chan, Rebecca Shaw, Gretchen Daily, and colleagues present a strategy for integrating ecosystem services into biodiversity conservation plans in California’s Central Coast ecoregion to systematically identify priorities for conservation.

Naidoo and Ricketts assessed five ecosystem services—sustainable bushmeat harvest, sustainable timber harvest, pharmaceutical bioprospecting, existence value (the intrinsic value of unspoiled wilderness), and carbon storage (forest conversion releases carbon dioxide)—provided by forests in the Mbaracayu Biosphere Reserve. The reserve—once covered by 90% forest but now highly fragmented and threatened beyond a protected core—supports large-scale cattle ranching, soybean production, and small-scale farming, along with hunting and foraging by the indigenous Ache.

In a previous study (co-authored by Naidoo), the opportunity costs of conserving forested land had been estimated by integrating expected agricultural production values with the probability of forests being converted to agriculture; the latter was calculated based on past patterns of deforestation. This procedure provided an estimate of the opportunity costs of conservation for each hectare of forest in the reserve.

To calculate conservation benefits, Naidoo and Rickets determined the beneficiaries and value of each ecosystem service per forest parcel across six forest types. There is no market price for bushmeat because it cannot be legally traded, so the authors calculated its value in part by multiplying the local price of a kilogram of store-bought beef (US$1.44) times the expected meat production (from 12 wild game species) for each forest hectare. Sixteen tree species in the reserve were used to estimate the average value of marketable timber per standing tree (US$6.87), assuming a sustainable harvest. Bioprospecting value was calculated based on drug companies’ willingness to pay for potentially marketable forest-derived drugs. Existence value was estimated based on various groups’ willingness to pay for forest preservation. And carbon storage value was calculated based on prices in the carbon-trading market (where companies get paid for stemming carbon dioxide emissions).

**Figure:**
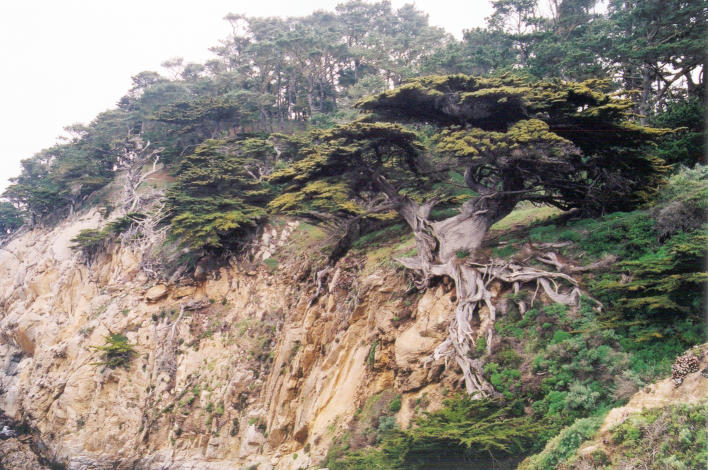
The moist forests of Point Lobos State Reserve and the northern Santa Lucia Range are important for biodiversity, carbon storage, flood control, recreation, and water provision. (Photo: Kai Chan)

Both the costs and the benefits of forest conservation varied considerably across this relatively small landscape. Interestingly, bioprospecting—long invoked to justify conserving areas that could harbor potentially life-saving medicines—offered the smallest benefit. When only local (bioprospecting, bushmeat, timber) services were included in the analyses, just a few parcels passed the cost-benefit test for conservation. But when carbon values were added—“by far” the most valuable service on average per hectare—benefits exceeded opportunity costs for 98% of the forests. Demonstrating how this approach could guide conservation planning, the authors show that linking two large forest patches with one wildlife corridor would provide far greater net benefits than either of two alternative corridors.

Building on recent advances in the emerging field of ecosystem service conservation, Chan et al. present an analytical framework that integrates ecosystem services into the well-established methods of biodiversity planning. Innovative “payment for environmental services” schemes, in which people who benefit from ecosystem services pay for them, have been introduced on a small scale in Guatemala, Indonesia, and other countries (and on a larger scale in Costa Rica). But expanding on these local programs requires a comprehensive, quantitative planning framework that links ecosystem services with biodiversity conservation. Toward that end, Chan et al. mapped the intersection between areas that provide ecosystem services and those that support substantial biodiversity. By modeling the implications of conservation plans based on prioritizing various combinations of biodiversity and ecosystem services—identifying tradeoffs in some cases and synergies in others—they illustrate the importance of integrating both elements in conservation planning.

**Figure:**
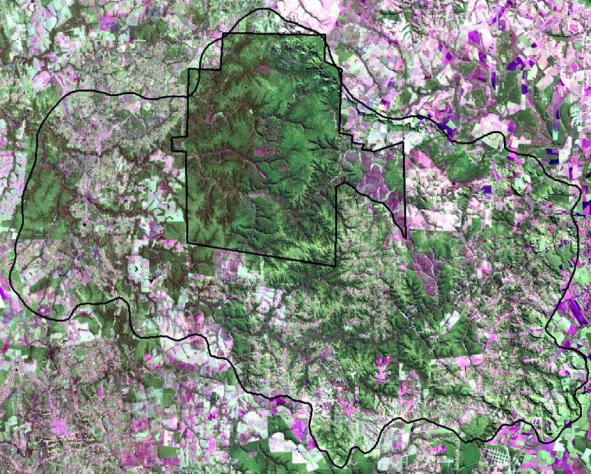
Landsat satellite image of the Mbaracayu Forest Biosphere Reserve. Forests (dark green) are steadily being converted to pastures, croplands, and industrial soybean farming. Economic arguments for deforestation usually ignore the important ecosystem services that such forests provide. (Image: Laura Rodriguez, Juan Viera, and Robin Naidoo)

The authors studied the California Central Coast ecoregion, covering north of San Francisco to Santa Barbara, where they had sufficient empirical and spatially explicit datasets for six ecosystem services provided by the 11,000-plus planning units in the region. They mapped terrestrial biodiversity and six services—carbon storage, flood control, forage (grazing) production, outdoor recreation, crop pollination, and water provision—across the region, and estimated each parcel’s provision of each service (following the same protocol used to plan biodiversity conservation) to develop conservation area networks for each service and biodiversity.

With a conservation planning software program, they mapped the spatial correlations between benefits (biodiversity and the six services) across parcels. They also quantified ecosystem services provided by a network of lands prioritized for biodiversity versus multiple ecosystem services. The spatial distribution of benefits from biodiversity and each of the six ecosystem services varied considerably across the ecoregion. Some mountain regions, for example, had high values for carbon storage, water provision, and recreation, while the agricultural Salinas Valley had high pollination service values and a small riparian area that provided flood control but low values for other services. Overall, spatial correlations between the ecosystem services were low (aside from a few high correlations, such as between carbon storage and water provision), as was the correlation between biodiversity and the other services. The authors found that there were generally low levels of overlap between planning units prioritized for different services.

The authors also determined tradeoffs between plans based on biodiversity versus ecosystem services by analyzing four different combinations of biodiversity/ecosystem networks; they also determined potential side benefits of adding ecosystem services to a biodiversity network. When biodiversity networks were considered as a whole, they found that “impressive supplies of ecosystem services” would be protected. Plans that prioritized networks based on ecosystem services—either with or without biodiversity, or a “strategic” set, with biodiversity but without the two agriculture-based services—protected slightly higher levels of services but lower levels of biodiversity. The strategic network performed well for biodiversity and had the best overall levels of all benefits. These results reveal important potential tradeoffs between conservation for biodiversity versus ecosystem services, underscoring the need for a systematic conservation planning framework that strategically targets the unique complement of features in a given region.

Understanding the degree of overlap between lands that provide important ecosystem services as well as biodiversity, Chan et al. argue, will not only reveal hotspots for conservation but also suggest new partners for ecosystem protection. It also provides an opportunity to build common ground between wilderness advocates and landowners to develop conservation initiatives with multiple winners. By the time hurricane Katrina hit, Louisiana had lost 405,000 hectares of wetlands—offering a bitter lesson on wetlands flood protection. Now, a growing list of industries sees wetlands restoration as the key to economic recovery in the area.

Together, these two studies contribute valuable analytical frameworks to the nascent field of studying and planning for ecosystem services. And by systematically identifying tradeoffs and opportunities for aligning plans to protect biodiversity with those to conserve the flow of services from an ecosystem, they provide policymakers with a decision-making framework to identify conservation hotspots and maximize the allocation of scarce conservation dollars.

